# Evidence of unconventional superconductivity on the surface of the nodal semimetal CaAg_1−*x*_Pd_*x*_P

**DOI:** 10.1038/s41467-023-42535-5

**Published:** 2023-10-26

**Authors:** Rikizo Yano, Shota Nagasaka, Naoki Matsubara, Kazushige Saigusa, Tsuyoshi Tanda, Seiichiro Ito, Ai Yamakage, Yoshihiko Okamoto, Koshi Takenaka, Satoshi Kashiwaya

**Affiliations:** 1 Department of Applied Physics, Nagoya University, Furo-cho, Chikusa-ku, Nagoya, 464-8603 Aichi Japan; 2 Department of Physics, Nagoya University, Furo-cho, Chikusa-ku, Nagoya, 464-8603 Aichi Japan; 3 Present Address: Institute for Solid State Physics, the University of Tokyo, Kashiwanoha 5-1-5, Kashiwa, 277-8581 Chiba Japan

**Keywords:** Superconducting properties and materials, Topological insulators

## Abstract

Surface states of topological materials provide extreme electronic states for unconventional superconducting states. CaAg_1−*x*_Pd_*x*_P is an ideal candidate for a nodal-line Dirac semimetal with drumhead surface states and no additional bulk bands. Here, we report that CaAg_1−*x*_Pd_*x*_P has surface states that exhibit unconventional superconductivity (SC) around 1.5 K. Extremely sharp magnetoresistance, tuned by surface-sensitive gating, determines the surface origin of the ultrahigh-mobility “electrons.” The Pd-doping elevates the Fermi level towards the surface states, and as a result, the critical temperature (*T*_c_) is increased up to 1.7 K from 1.2 K for undoped CaAgP. Furthermore, a soft point-contact study at the surface of Pd-doped CaAgP proved the emergence of unconventional SC on the surface. We observed the bell-shaped conductance spectra, a hallmark of the unconventional SC. Ultrahigh mobility carriers derived from the surface flat bands generate a new class of unconventional SC.

## Abstract (web summary)

CaAg_1−*x*_Pd_*x*_P is a nodal-line Dirac semimetal. Here, using ionic-liquid gated transport and soft point-contact spectroscopy, the authors show that this material realizes surface-confined unconventional superconductivity.

## Introduction

Non-trivial surface states of topological materials provide sources of exotic electronic conditions; for example, linear-band dispersion leads to ultrahigh-mobility carriers, and spin-polarized states induce enriched magnetic phenomena^1–4^. Such spin-polarization, mainly derived from strong spin-orbit coupling (SOC), forms unconventional Cooper pairing, such as chiral *p*-wave superconductivity (SC). Several unconventional SC can host Majorana quasiparticles, whose antiparticles are themselves^5^. On the other hand, strong on-site repulsive interactions drive the anisotropic wave function of Cooper pairs yielding unconventional SC^6,7^. Thus, several experimental efforts to realize unconventional SC have been performed in materials possessing flat bands, e.g., bilayer graphene^8^. Such materials potentially lead to high critical temperature (*T*_c_) exceeding room temperature and spin-triplet SC^9,10^. Namely, extreme conditions associate with unconventional SC. Therefore, combining the flat band and extreme conditions provided by topological surface states without strong SOC may provide a new class of unconventional SC.

**Fig. 1:**
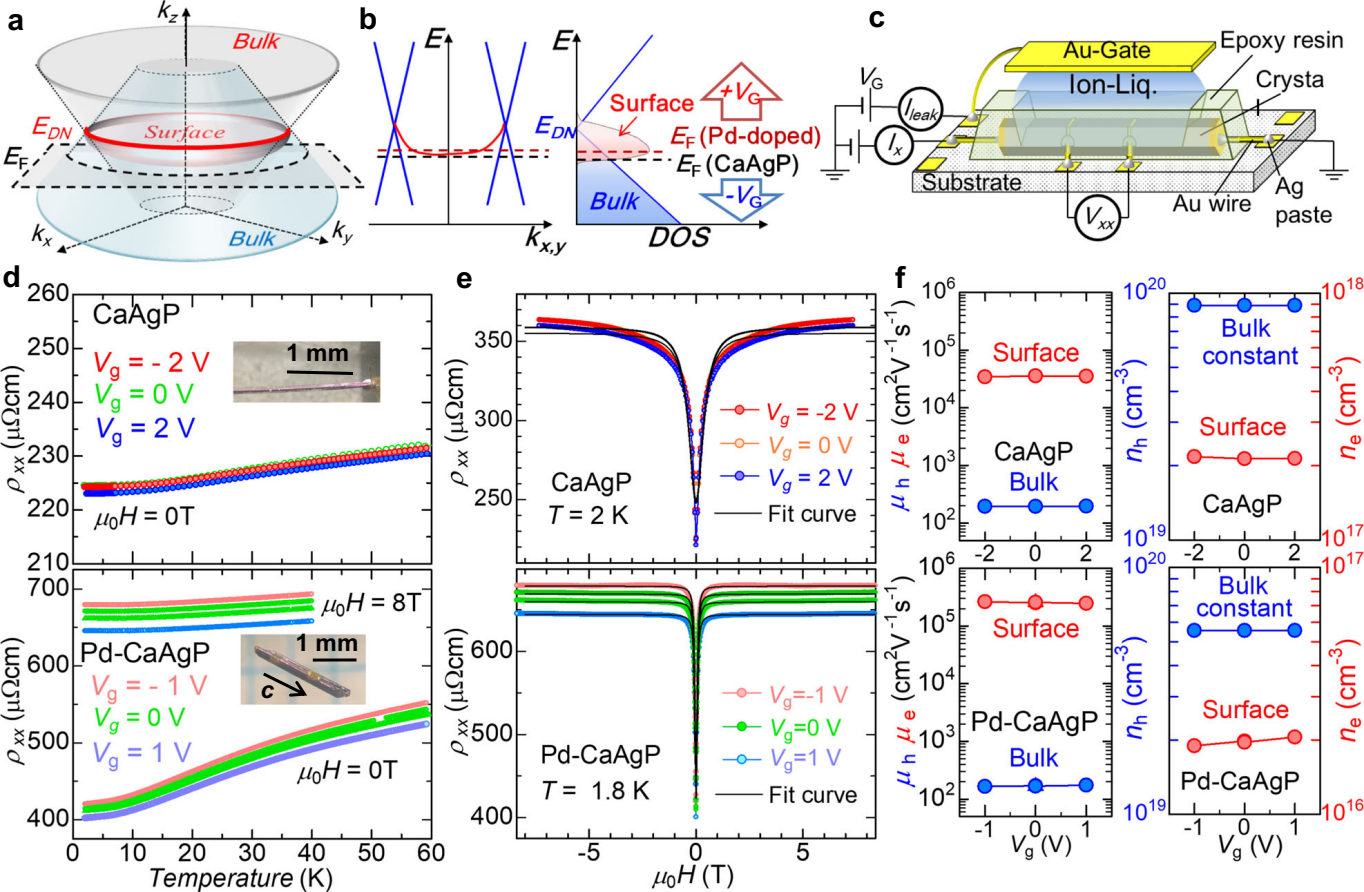
Gate-tuned transport properties of undoped and Pd-CaAgP. **a** Schematic band structures of CaAgP. The red circle represents the nodal line (ring) at which Dirac-like dispersion crosses at ${E}_{{{{{{{{\rm{DN}}}}}}}}}$. The Fermi level is located just below ${E}_{{{{{{{{\rm{DN}}}}}}}}}$. Drumhead-like surface states appear within the ring. **b** Schematic energy dispersion and the DOS of bulk states (blue) and surface states (red) of CaAgP. The dashed lines are the Fermi levels for undoped (black) and Pd-doped (red) CaAgP. A positive (negative) gate voltage shifts up (down) the chemical potential indicated by the red (blue) arrow. **c** Schematic sample configuration for IL-gating transport measurements. **d** Temperature dependence of gate-controlled CaAgP (upper) for *V*_*g*_ = −2 (red), 0 (green), and 2 V (blue) and Pd-CaAgP (bottom) for Vg = −1 (pink), 0 (green), and 1 V (cyan blue) at zero-field and 8 T. The measurement order for CaAgP (Pd-CaAgP) was 0V– -2V–2V (1V–0V– -1V–0V). The insets are photos of the crystals. **e** Magnetoresistivity for CaAgP (upper) and Pd-CaAgP (bottom) under the gate-control. Black lines are fitting results by the two-band model, with the bulk carriers remaining unchanged by the gate voltage. **f**, left panel The estimated mobility and (**f**, right panel) the carrier density. The upper (lower) panels are CaAgP (Pd-CAgP). The parameters were deduced by using the two-band model.

Here, we focus on CaAgP, a candidate nodal-line semimetal with a unique nodal line (ring) where linear bulk bands cross owing to weak SOC (Fig. 1a)^11^. Surface states are expected to exist in the nodal ring, forming drumhead dispersion in the (0 0 1) plane^11^. The bottom of the drumhead surface can be regarded as a flat band (Fig. 1b). The density of states (DOS) of the surface states is concentrated at the flat band below the Dirac nodal-ring energy ${E}_{{{{{{{{\rm{DN}}}}}}}}}$, as shown in Fig. 1b. Reflecting this unique band structure, CaAg_1−*x*_Pd_*x*_P has ultrahigh mobility carriers^12^. By the combination of the extreme electronic structure and flat bands that provide extremely large DOS and strong interactions, we expect the drumhead surface states to exhibit unconventional surface SC^13,14^. Furthermore, previous work discovered two unusual features of Pd-doped CaAgP (Pd-CaAgP)—unusually sharp magnetoresistance and SC at 1.7 K without any heat-capacity anomaly around *T*_c_^12^—which motivated us to investigate their surface electronic states.

In this study, we use systematic three experiments to demonstrate that CaAgP has exotic surface states that show unconventional SC: (i) surface-sensitive transport measurements using gate-voltage (*V*_g_) control through an ionic liquid (IL), (ii) low-temperature SC measurements at 0.6–3 K, and (iii) soft point-contact measurements to investigate the SC states of Pd-CaAgP. We report direct evidence that “electrons” dominate the surface that shows SC. Undoped CaAgP exhibits SC at 1.2 K, and Pd-doping elevates *T*_c_ up to 1.7 K. The soft point-contact study provides smoking-gun evidence of unconventional surface SC in Pd-CaAgP.

Gate-tuning uses an IL to form an electronic double layer adjacent to the surface, generating a strong electric field within a few nanometers from the surface. The decay length of this electric field is typically inverse to the carrier density, enabling us to tune a wide range of carrier densities just below the interface of a metallic system. Figure 1c illustrates the measurement configuration using IL gating. We evaluated the transport properties in the (1 0 0) planes of hexagonal, needle-like CaAg_1−*x*_Pd_*x*_P crystals (see Supplementary Fig. S1). Figure 1d shows the temperature dependence of the resistivity of undoped CaAgP and Pd-CaAgP at different gate voltages; the general trends are consistent with the previous work^12^. While undoped CaAgP is less affected by *V*_*g*_, Pd-CaAgP shows gate-dependent *ρ*−*T* behavior below 60 K. Positive voltages provide electrons to the surface, decreasing the resistivity below 60 K, while negative voltages have the opposite effect.

Figure 1e shows that the resistivity of both crystals rapidly increased to specific saturation values by applying a magnetic field. The gated transports for CaAgP (Pd-CaAgP) were measured in the order 0V– −2V–2V (1V–0V– −1V–0V) to distinguish the *V*_*g*_ dependence and cycle dependence such as the deterioration and chemical reactions. Magnetoresistivity (*ρ*_*xx*_(*H*)) seems prominent by the Pd-doping (Fig. 1e). Several topological materials such as MoTe_2_^15^, TaAs^16^, and FeSb_2_^17^ show a similar rapid increase in *ρ*_*xx*_(*H*), which can be understood using a typical two-band model^18^. This model enables us to evaluate mobility and carrier density. The sharp response of magnetoresistivity indicates the existence of an ultrahigh-mobility carrier (see ref. ^19^ and Supplementary Fig. S2).

Figure 1f summarizes the results obtained by fitting the gate-controlled magnetoresistance. Details of the fitting procedures are presented in Supplementary Section 2, and the validity of the gating and cycle dependence are described in Supplementary Section 3. The carrier density of CaAg_1−*x*_Pd_*x*_P is low as metal and comparable with that of the low-carrier-density superconductors (Tl-doped PbTe with ${T}_{{{{{{{{\rm{c}}}}}}}},\max } \sim$1.5 K has ~10^20^ cm^−3 ^^20^). The “electron” carriers of Pd-CaAgP pronouncedly responded to the gate voltage, which indicates that the applied voltage works almost exclusively at the surface, where the dominant carriers are “electrons.” This sheet electron density is *n*_e,2D_ ~ 10^11^ cm^−2^ assuming the surface thickness of several nanometers. The evaluated value is consistent with the surface carrier density obtained from the quantum oscillations, as described later.

On the other hand, the bulk carriers are holes, the density of which decreases with Pd-doping, which is also supported by the lattice shrinkage caused by doping (see Fig. S1). Thus, Pd-doping elevates the Fermi level, *E*_F_, towards the bottom of the surface band (Fig. 1b). As a result, IL gating effectively tunes only the surface electrons for Pd–CaAgP. Therefore, these gating experiments support the existence of surface states. Note that the present measurement probes the (1 0 0) plane, while theory predicts the emergence of the drumhead surface in the (0 0 1) plane. This discrepancy suggests that additional topologically protected surface states may exist at the side planes or the as-grown non-flat measurement planes may include a projected component from the (0 0 1) plane.

**Fig. 2:**
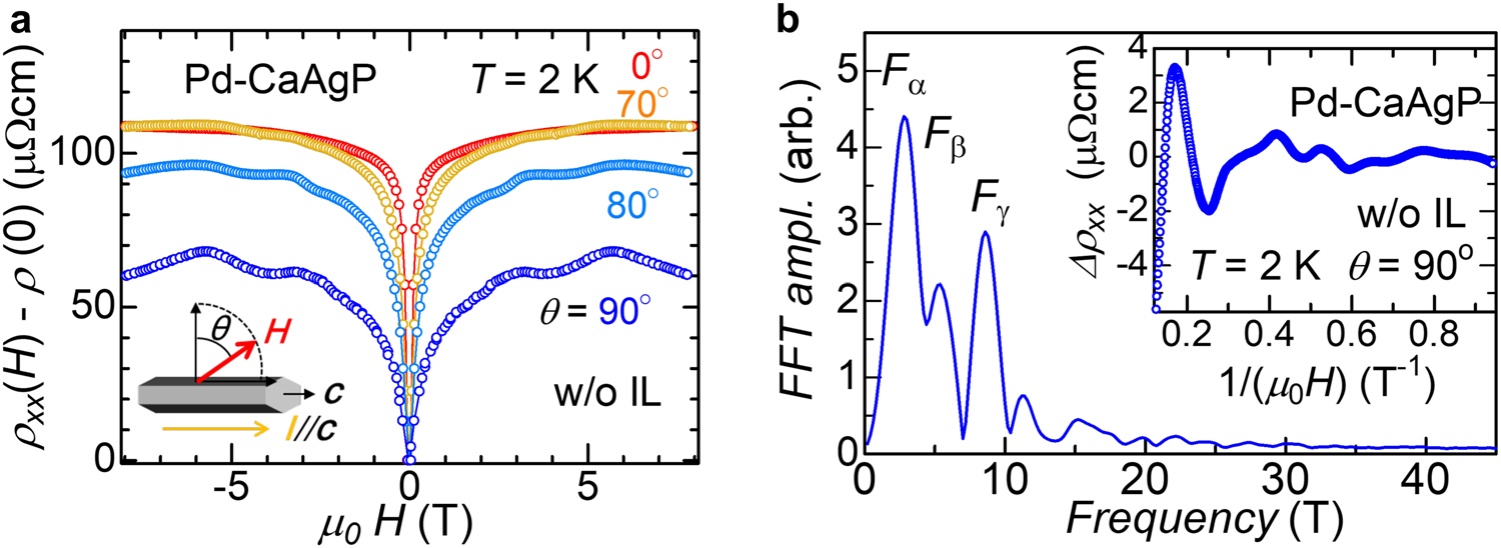
Quantum oscillation analysis of Pd-CaAgP. **a** Field dependence of magnetoresistance of Pd-CaAgP at several magnetic field directions (No gate voltage applied). Inset indicates schematic sample configuration between a magnetic field and current flow direction. **b** FFT spectrum of oscillations in magnetoresistance at *θ* = 90^∘^ after subtracting two-band magnetoresistance contributions (Inset) without no-gate voltage at 2 K. Three peaks are labeled as *F*_*α*_, *F*_*β*_, and *F*_*γ*_ from smaller frequency. We confirmed that different windows also produce finite three peaks (supplementary Fig. S8).

To determine the Fermi surface, we applied a large magnetic field and varied angles between the field and the *c*-axis without IL gating (Fig. 2a). Above 2 T and around *θ* = 90°, the resistivity of Pd-CaAgP shows Shubnikov de Haas (SdH) oscillations, which reflect the Landau-level separation perpendicular to the magnetic field. The oscillations observable at such low fields are associated with ultrahigh-mobility carriers. Typical fast Fourier transformation (FFT) processing of oscillatory components, extracted from fitting the conventional magnetoresistance components (the inset in Fig. 2b), revealed three oscillatory components (Fig. 2b). Despite the difficulty of exact peak identification under the limited magnetic field (~9 T), these peaks have only low frequencies of less than 10 T, indicating that the Fermi surface is tiny. From the Onsager relation, *F*_*α*,*β*,*γ*_ corresponds to *k*_*α*,*β*,*γ*_ = 0.0092, 0.013, and 0.016 Å^−1^, respectively. These low values ensure that *E*_F_ is located at the bottom of the drumhead surface below ${E}_{{{{{{{{\rm{DN}}}}}}}}}$. These values are comparable to typical values for nodal-line semimetals (9 T for ZrSiS^21^) with ${E}_{{{{{{{{\rm{F}}}}}}}}} \sim {E}_{{{{{{{{\rm{DN}}}}}}}}}$. However, the drumhead surface produces complex Fermi surfaces^22^. Assuming an ideal circular Fermi surface, *k*_*α*,*β*,*γ*_ corresponds to a carrier density of *n*_2D_ ~ 10^11^ cm^−2^, which is close to *n*_e,2D_ of the two-band model results. Furthermore, the angular dependence of the FFT peaks follows 1/cos*θ*, a hallmark of two-dimensional electronic states (Supplementary Fig. S8). Thus, these peaks originate from the surface states, not from the bulk states.

One possible interpretation of these three peaks is that they may arise from different planes of the hexagonal, needle-like crystal and belong to the same *k*_*γ*_. Other nodal-line semimetals^21,23^—and the similar analog compound CaAgAs^24^, which become a topological insulator due to the strong spin-orbit coupling of As—typically have high-frequency components above 100 T that come from the bulk bands, without such low-frequency contributions. Despite our limited magnetic-field strengths, the SdH oscillations we observed support the emergence of surface states, with *E*_F_ located just below ${E}_{{{{{{{{\rm{DN}}}}}}}}}$.

**Fig. 3:**
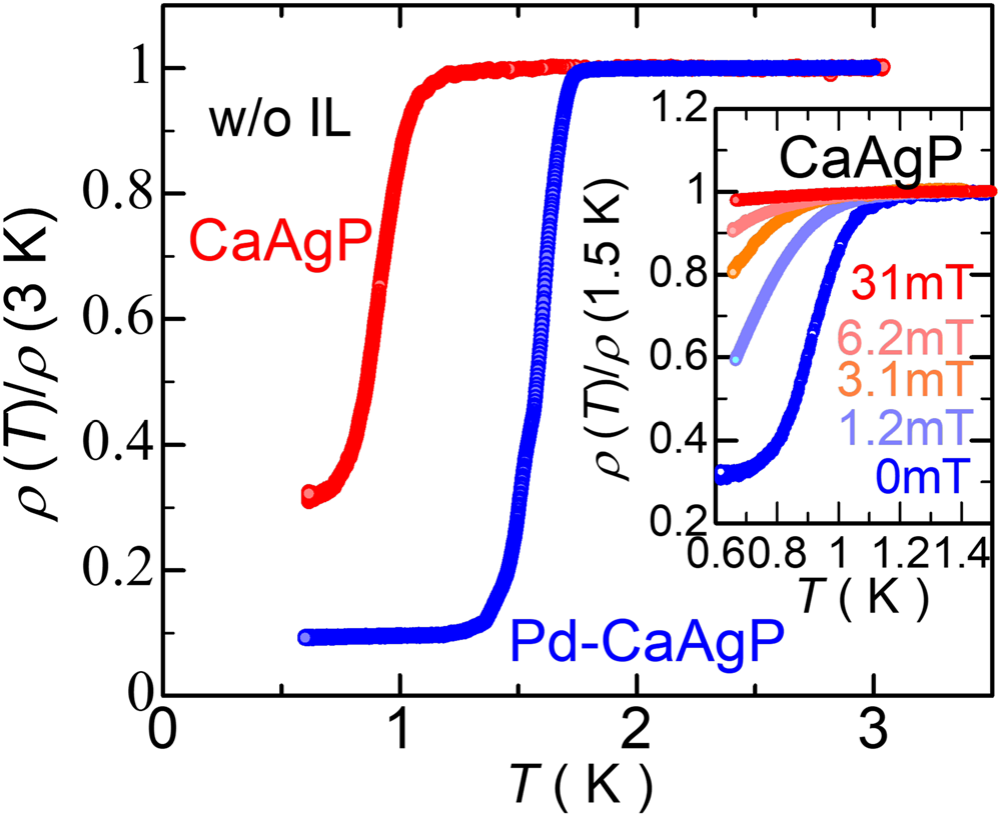
Superconducting properties of undoped and Pd-doped CaAgP. Temperature dependence of resistance normalized at 3 K in undoped and Pd-CaAgP crystals. Inset shows temperature dependence of normalized resistance under several magnetic fields. The zero resistance was not observed due to the high sensitivity to the sensing current, as discussed in Supplementary Section 6.

We performed low-temperature measurements using a ^3^He-refrigerator to evaluate the SC of CaAg_1−*x*_Pd_*x*_P without using IL gating. In addition to Pd-CaAgP with *T*_c_ of 1.7 K^12^, we found that undoped CaAgP exhibits SC around *T*_c_ ~ 1.2 K (Fig. 3a). This SC state is easily broken by a small magnetic field (~30 mT) and a small measurement current (the inset in Fig. 3 and Supplementary Fig. S9). While Pd-doping decreases both hole and electron carrier density, the elevated *E*_F_ level enhances condensation of the surface electrons that may increase *T*_c_. The present surface carrier density *n*_e,2D_ ~ 10^11^ cm^−2^, is much smaller than the critical carrier density of a 2D SC, e.g., 1.7 × 10^13^ cm^−2^ for LAO/STO^25^. Thus, only spatially condensed electrons at the surface exceed critical density, forming surface SC. Indeed, a previous study showed that a visible anomaly does not accompany the SC transition in Pd-CaAgP in the specific heat capacity^12^, which is consistent with the present surface SC picture.

Furthermore, the surface flat-band may induce an unconventional SC. We estimated the ratio of *T*_c_ and the effective Fermi temperature *T*_F_ to locate the position of the surface SC in the Uemura plot^26^. The *T*_F_ is calculated by

1 $${T}_{{{{{{{{\rm{F}}}}}}}}}=\frac{(\pi {\hslash }^{2}){n}_{2{{{{{{{\rm{D}}}}}}}}}}{{k}_{{{{{{{{\rm{B}}}}}}}}}{m}^{*}}$$

for 2D systems. The conventional superconductors following the Bardeen-Cooper-Schrieffer (BCS) theory have *T*_c_/*T*_F_ ~ 10^−5^ (which corresponds to *n*_2D_/(m^*^/*m*_*e*_) ~ 10^15^ cm^−2^ at *T*_c_ ~ 1 K), while cuprates and other unconventional superconductors *T*_c_/*T*_F_ ~ 10^−2^ (*n*_2D_/(m^*^/*m*_*e*_) ~ 10^12^ cm^−2^ at *T*_c_ ~ 1 K)^26^. Considering to similar compounds, such as nodal-line semimetal ZrSiS (the cyclotron effective mass of the surface carriers m^*^ = 0.15*m*_*e*_^21^) and a family compound CaCdGe/CaAgAs (m^*^ = 0.23*m*_*e*_^27^/0.13*m*_*e*_^24^), the ratio of *n*_e,2D_/(m^*^/*m*_*e*_) of CaAg_1−*x*_Pd_*x*_P is expected to be ~10^12^ cm^−2^, suggesting unconventional SC.

The 2D nature of the surface SC is supported by the Berezinsky-Kosterlitz-Thouless (BKT) transition in the voltage-current curves^28,29^. The *V*–*I* curves show a non-Ohmic behavior around *T*_BKT_, where the *V*-*I* curve depends on *V* ∝ *I* ^*α*^ with *α* = 3, according to the BKT transition scenario. As discussed in Supplementary Fig. S10, we determined the BKT temperature of 1.05 K. A similar transition is widely observed in 2D SC, such as magic angle bilayer graphene^30^. Thus, those facts also support the emergence of the surface SC on the Pd-CaAgP.

**Fig. 4:**
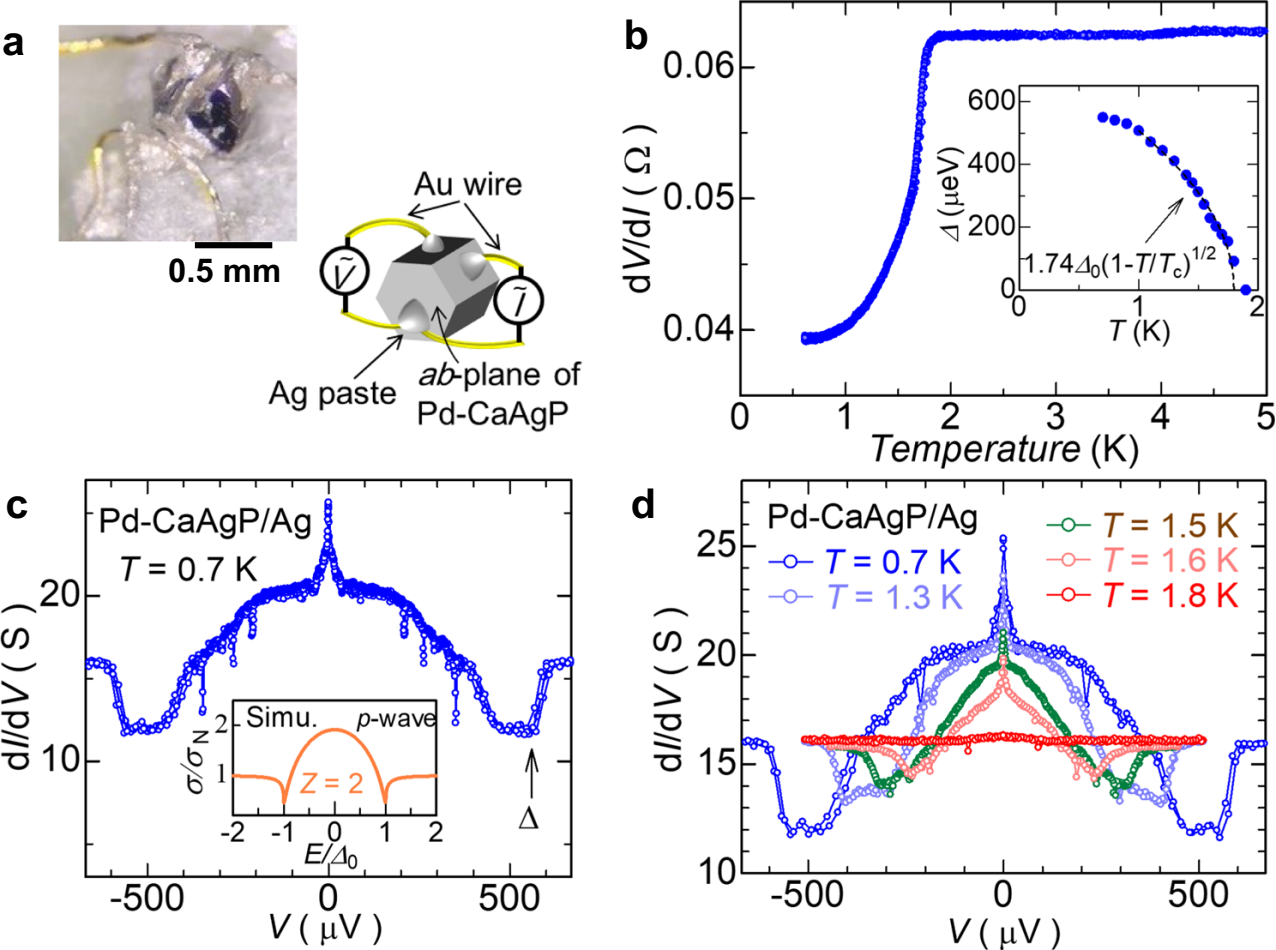
Soft point contact results of Pd-CaAgP. **a** Schematic and photo of sample configurations. Note that, the measurement spot was (0 0 1) plane. **b** Temperature dependence of differential resistance with zero bias voltage. Inset indicates temperature dependence of estimated superconducting gap functions, Δ, determined by dips of the spectra. The value of Δ_0_ = 437 *μ*eV was obtained by the fitting. **c** Differential conductance spectrum of Pd–CaAgP/Ag junction at 0.7 K. The gap function was determined at intersection point of two dashed lines. Inset shows calculated spectrum for *p*-wave SC. **d** Temperature evolution of the conductance spectra of Pd-CaAgP/Ag junction.

We investigated the surface SC by soft point contact technique, a surface-sensitive measurement that can identify the SC gap structure. Figure 4a illustrates the measurement configuration we used to probe the (0 0 1) plane of Pd-CaAgP. The temperature dependence of the differential resistivity shows a clear SC transition at 1.7 K. At low temperatures, the differential-conductance peaks exhibited an unconventional bell-like shape and a sharp zero-bias peak (Fig. 4c), while typical *s*-wave SC exhibits a gap structure (Supplementary Fig. S11). Spiky dips are often observed in microjunctions with inhomogeneous barrier contacts^31^. The sharp zero-bias peak may represent Majorana bound states (details are in Supplementary Section 7).

Because a spurious ZBCP can emerge when the point-contact junction is in the thermal regime^32^, we rejected the influences from the heating effect for several reasons. One of the essential facts is that the applied bias current density (– ~ 5 A/cm^2^) is several orders more smaller than the estimated critical current density (~10^3^ A/cm^2^). Other discussions related to the heating effect are provided in Supplementary Section 7.

The temperature dependence of the gap function Δ determined at the outermost dips shows a typical BCS-like dependence around *T*_c_,

2 $$\Delta=1.74{\Delta }_{0}\sqrt{1-T/{T}_{{{{{{{{\rm{c}}}}}}}}}}.$$

By the fitting, we obtained Δ_0_ = 437 *μ*eV and 2Δ_0_/*k*_B_*T*_c_ = 5.97, which is larger than the conventional value of 3.52, suggesting the existence of a strong electron correlation. Figure 4d plots the temperature evolution of the conductance spectra. Interestingly, the sharp zero-bias conductance peak (ZBCP) appears accompanied by the emergence of the bell-like in-gap states below 1.7 K.

While the barrier height at the contact interface typically affects the conductance spectra, a conventional SC does not accompany the dips at Δ (Supplementary Fig. S11). However, a bell-shaped spectrum is predicted theoretically for spin-triplet *p*-wave SC states within an extended Blonder–Tinkham–Klapwijk (BTK) formula^33–35^ (the inset in Fig. 4); it represents an Andreev bound state that provides smoking-gun evidence for unconventional SC as well as the emergence of the surface SC.

Flat-band SC has recently been discussed for twisted bilayer graphene^8^. This flat band is realized by heterostructure engineering, and their unique moiré potential contributes to the formation of *p*-wave SC. On the other hand, protected flat surface states of CaAgP may provide both strong electron interaction and extreme conditions such as ultrahigh-mobility carriers. The combination of such a flat band and the extreme conditions may provide a new class of exotic SC states. Indeed, one theory predicts the emergence of unconventional surface SC depending on bulk SC states and the chemical potential^14^.

Furthermore, unlike the bilayer systems, the surface states of CaAgP are determined by bulk crystal symmetry and are expected to be robust against surrounding conditions. Such surface states show spontaneously surface SC without additional operations such as doping and pressure effects. CaAg_1−*x*_Pd_*x*_P does not contain any hazardous and radioactive elements. Therefore, accessibility to this new class of unconventional SC in CaAg_1−*x*_Pd_*x*_P may facilitate investigating these curious electronic SC states derived from flat bands and topological physics, indicating that— like cuprates—CaAg_1−*x*_Pd_*x*_P is likely to become a leading SC material in condensed matter physics.

## Methods

### Sample preparation

We grew crystals of CaAg_1−*x*_Pd_*x*_P (nominal *x* = 0 and 0.1) using a Bi-flux method^12^, removing excess Bi-flux by centrifugation at 673 K. Appropriated raw materials in an alumina crucible sealed in a silica tube are slowly cooled from 1273 K at the rate of −12 Kh^−1^. We checked crystal phases and crystal orientation at room temperature using powder-XRD equipment (MiniFlex by Rigaku Corp.).

### Gate-tuned transport measurements

We evaluated the transport properties of bulk single crystals over the range of 2–300 K with the Physical Property Measurement System (Quantum Design Japan, Inc), using the R6244 source/current monitor (Advantest Corp.) to apply external gate-voltage and monitor current leakage. Details of sample preparation for the IL-gating configuration are provided in ref. ^36^. We created ohmic contacts between the Au wires and the crystals using Ag paste, and we covered the Ag with epoxy resin to prevent corrosion by the IL (Fig. 1c). We symmetrized the magnetoresistance data by averaging them over positive and negative magnetic fields to obtain the even-function resistivity. We applied the gating slowly at 240 K to prevent chemical reactions with and consolidation of the IL (the glass temperature is about 200 K; DEME-TFSI (Kanto Chemical Co., Inc.)). After cooling the sample down to 40 K, we turned off the gate voltage. We performed all transport measurements of Pd-CaAgP below 60 K to avoid the abovementioned adverse effects. We repeated the transport measurement for several sweep cycles to distinguish the gate dependence and cycle dependence.

### Low-temperature and soft-point contact study

We performed the low-temperature resistance and soft point-contact measurements over the range of 0.6–3 K using a ^3^He refrigerator. We measured the differential conductance of the Pd-CaAgP/Ag junctions using a standard lock-in technique with a modulation frequency of 371.1 Hz.

### Supplementary information

Supplementary Information

## Data Availability

The data that support the findings of this study are available in figshare (10.6084/m9.figshare.24088626). Additional data are available from the corresponding authors upon reasonable request.
