# Supplementary material for: Evidence of unconventional superconductivity on the surface of the nodal semimetal CaAg1−xPdxP
Source: Nat Commun. 2023 Oct 26;14:6817. doi: 10.1038/s41467-023-42535-5 (PMC10603147; doi:10.1038/s41467-023-42535-5)
Supplement: Supplementary file 1 — Supplementary Information [file 41467_2023_42535_MOESM1_ESM.pdf]

Supplementary Information for Evidence of Unconventional Superconductivity on the Surface of the Nodal Semimetal $CaAg_{1-x}Pd_xP$

Rikizo Yano[1*], Shota Nagasaka[1], Naoki Matsubara[1], Kazushige Saigusa[1], Tsuyoshi Tanda[1], Seiichiro Ito[1], Ai Yamakage[2], Yoshihiko Okamoto[1,3*], Koshi Takenaka[1] and Satoshi Kashiwaya[1*]

[1]Department of Applied Physics, Nagoya University, Furo-cho, Chikusa-ku, Nagoya, 464-8603, Aichi, Japan.
[2]Department of Physics, Nagoya University, Furo-cho, Chikusa-ku, Nagoya, 464-8603, Aichi, Japan.
[3]Current Institution: Institute for Solid State Physics, the University of Tokyo, Kashiwanoha 5-1-5, Kashiwa, 277-8581, Chiba, Japan.

*Corresponding author(s). E-mail(s): yano-rikizo@nagoya-u.jp; s.kashiwaya@nagoya-u.jp;

Supplementary section 1:XRD and crystal structure

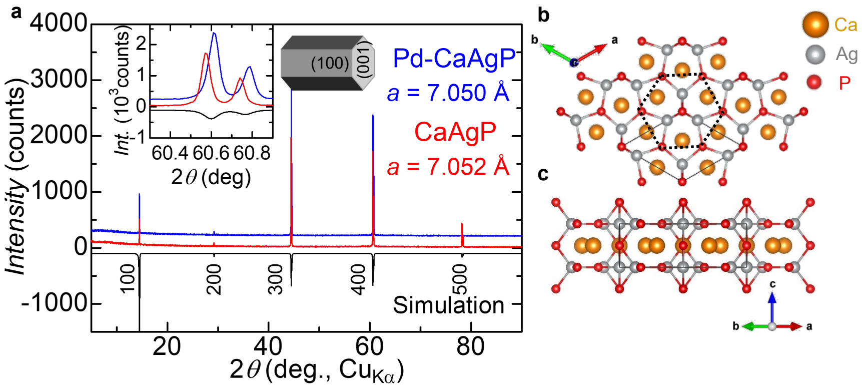

Fig. S1 Crystal structure and XRD analysis of $CaAg_{1-x}Pd_xP$. a, XRD patterns from (h 0 0) planes of needle-like crystals of $CaAg_{1-x}Pd_xP$. The black line represents a simulated pattern of undoped CaAgP. Inset is highlighted patterns of 4 0 0 peaks. Lattice parameters of $CaAg_{1-x}Pd_xP$ are a = 7.052 and 7.050 Å for nominal x = 0, and 0.1, respectively. **b,c,** Crytstal structures of CaAgP from top view (b) and side view (c).

The obtained crystals have a hexagonal needle-like shape, reflecting their crystal symmetry (The crystal structure is displayed in Fig. S1(b)and(c)). XRD patterns of the side plane of the hexagonal needle crystals $CaAg_{1-x}Pd_xP$ are described in Fig. S1(a). Only h 0 0 peaks were observed, indicating that the side planes are assigned by (1 0 0). The peaks were slightly shifted toward higher-angle by the Pd-doping. This shift indicates the lattice is shrinking. Assuming the ionic model ($Ca^{2+}Ag^{+}P^{-3}$ with Ag^{+} (1.00 Å) and Pd^{2+} (0.86 Å)), the most plausible interpretation of the Pd-doping on Ag-site with the Kröger-Vink notation is $Ag_{Ag} \rightarrow Pd^{\bullet}_{Ag} + e'$, leading to the lattice shrinking and electron-doping. This electron-doping is consistent with the present experimental results. On the other hand, other site-substitution (e.g., Ca-vacancy site: $Ca_{Ca} \rightarrow V''_{Ca} + 2h^{\bullet} + Ca(s)$) or Pd^{+0}-substitution (e.g., $Ag_{Ag} \rightarrow Pd'_{Ag} + h^{\bullet}$) lead to hole-doping. This is why we concluded that Pd substitutes for Ag-site and leads to electron-doping. Considering the actual composition of $x \sim 0.07$ for $CaAg_{1-x}Pd_xP$ and lattice parameters, Pd-doping produces 1.2×10^{20} cm^{-3}, which is comparable to the present carrier change by the doping calculated from the two-band model. Other groups also reported single crystals of CaAgP with plate-like crystal shapes grown by chemical vapor transport technique using I_2 as a transport agent [S1]. This group reported trivial surface states and bulk bands determined by ARPES measurements and DFT calculation [S1]. Different crystal morphology (our crystals have needle-like shape) implies the existence of different growth kinetics and different types of crystal defects that leads to lattice shrinking/expansion and chemical potential shift. Indeed, this group calculated band structure using $a = 7.1131$ Å [S1],

and the Fermi energy is drastically different from our results. Those discrepancies may affect band structure and surface topology. In fact, we confirmed that slight calculation condition differences, such as lattice constants, affect band dispersion around the Fermi energy.

Supplementary section 2:Simulations of the two-band model for $CaAg_{1-x}Pd_xP$

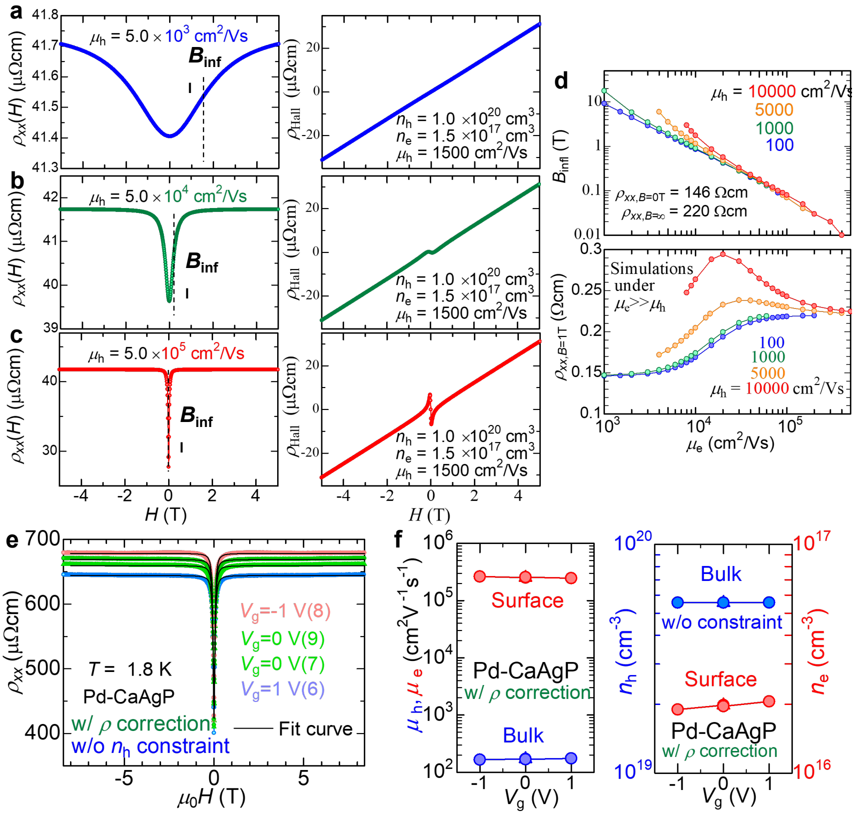

Fig. S2 Simulated magnetoresistance and Hall resistivity using the two-band model. a-c, Results of simulation with different electron mobility of $\mu_e = 5.00 \times 10^3$ (a), 5.00×10^4 (b), 5.00×10^5 cm^2/Vs (c). Other parameters are fixed to the experimentally determined values as provided bottom, right. Dashed lines represent inflection points $B_{\rm infl}$, characteristic points where ρ_{xx} rapidly increases to a saturation value. **d,** Simulation results of an inflection point $B_{\rm infl}$ for ρ_{xx} (upper panel) and ρ_{xx} at $B = 1$ T (bottom) under the condition of $\mu_e \gg \mu_h$ (resulting $n_h \gg n_e$). **e,** Magnetoresistivity for Pd-CaAgP under the gate-control. Black lines are fitting results by the two-band model without the n_h-constant constraint. **f,** Evaluated carrier density and mobility by the fitting without the n_h-constant constraint.

In the previous work [S2], Hall measurement was adopted to evaluate carrier density. The Hall resistance of Pd-CaAgP exhibited non-linear H-dependence, which points to the existence of both electrons and holes as carriers. On the other hand, undoped CaAgP showed a positive linear Hall resistivity, which indicates dominant hole carriers. It is difficult to applicate the two-band model for Hall resistivity of undoped CaAgP in this case. However, the two-band model has two components, that is, magnetoresistance and

Hall resistivity:

$$\rho_{xx} = \frac{1}{e}\frac{(n_{\mathrm{h}}\mu_{\mathrm{h}} + n_{\mathrm{e}}\mu_{\mathrm{e}}) + \mu_{\mathrm{h}}\mu_{\mathrm{e}}(n_{\mathrm{h}}\mu_{\mathrm{e}} + n_{\mathrm{e}}\mu_{\mathrm{h}})B^2}{(n_{\mathrm{h}}\mu_{\mathrm{h}} + n_{\mathrm{e}}\mu_{\mathrm{e}})^2 + (n_{\mathrm{h}} - n_{\mathrm{e}})^2\mu_{\mathrm{h}}^2\mu_{\mathrm{e}}^2B^2} \tag{1}$$

and

$$\rho_{yx}(B) = \frac{B}{e}\frac{((n_{\mathrm{h}}\mu_{\mathrm{h}}^2 - n_{\mathrm{e}}\mu_{\mathrm{e}}^2) + \mu_{\mathrm{h}}^2\mu_{\mathrm{e}}^2(n_{\mathrm{h}} - n_{\mathrm{e}})B^2)}{(n_{\mathrm{h}}\mu_{\mathrm{h}} + n_{\mathrm{e}}\mu_{\mathrm{e}})^2 + (n_{\mathrm{h}} - n_{\mathrm{e}})^2\mu_{\mathrm{h}}^2\mu_{\mathrm{e}}^2B^2} \tag{2}$$

We noticed that magnetoresistance ρ_{xx} is more suitable for evaluating carrier density and mobility in these materials. As shown in Fig. S2(a), even though linear Hall resistance of undoped CaAgP (ρ_{yx}), magnetoresistance (ρ_{xx}) rapidly increased by applying a magnetic field. This rapid increase becomes prominent when the mobility of electrons becomes enhanced (Fig. S2(a)–(c)).

In the fitting procedure, we adopt two parameters (n_{e} and n_{h}) as fitting parameters. We can obtain one constrained condition by resistivity at zero magnetic field:

$$\rho_{xx}(0) = \frac{1}{e}\frac{1}{n_{\mathrm{h}}\mu_{\mathrm{h}} + n_{\mathrm{e}}\mu_{\mathrm{e}}}. \tag{3}$$

Considering a high magnetic field limit, we can obtain a saturation value of

$$\rho_{xx}(\infty) = \frac{1}{e}\frac{\frac{n_{\mathrm{h}}}{\mu_{\mathrm{h}}} + \frac{n_{\mathrm{e}}}{\mu_{\mathrm{e}}}}{(n_{\mathrm{h}} - n_{\mathrm{e}})^2}. \tag{4}$$

If the condition of $\mu_{\mathrm{e}} \gg \mu_{\mathrm{h}}$ [leading to$n_{\mathrm{h}} \gg n_{\mathrm{e}}$ due to the $\rho_{xx}(0)$] is valid, the saturation value becomes a simple expression:

$$\rho_{xx,\mu_{\mathrm{e}} \gg \mu_{\mathrm{h}}}(\infty) = \frac{1}{e\mu_{\mathrm{h}}n_{\mathrm{h}}}, \tag{5}$$

and thus, the saturation value is dominated by the carrier density for "hole" carriers. This expression works as a second constrained condition. Figure S2 (d) shows simulation results of an inflection point for ρ_{xx} (upper panel) and ρ_{xx} at $B = 1$ T (bottom) under the condition of $\mu_{\mathrm{e}} \gg \mu_{\mathrm{h}}$ (resulting $n_{\mathrm{h}} \gg n_{\mathrm{e}}$). The inflection point of ρ_{xx} is a characteristic point where ρ_{xx} rapidly increases to a saturation value. We can confirm that the condition of $\mu_{\mathrm{e}} \gg \mu_{\mathrm{h}}$ leads to rapid saturation at a low magnetic field. When this condition was not validated, e.g., $\mu_{\mathrm{e}} \sim \mu_{\mathrm{h}} = 10000$ cm^2/Vs, ρ_{xx} increases resonantly, as shown in the bottom panel of Fig. S2 (d). In other words, we can obtain valid fitting results for $\rho_{xx}(B)$ by using the two parameters under the condition of $\mu_{\mathrm{e}} \gg \mu_{\mathrm{h}}$. Because surface carriers mainly depend on the gate voltage, ρ_{xx} (0T) changed with gate voltages, as shown in Fig. 1(d). On the other hand, the value of ρ_{xx} at a high magnetic field (saturation values) showed slight gate-dependence (Fig. S2 (e)), reflecting unchanged n_{h}.

The initial value and fitting conditions affect the two carrier model fitting results. The present study used Hall measurement results at a high field without IL for the starting value of the hole carrier. Initially, we used ρ(0T) and

ρ(8T) values to reduce variable parameters, avoiding falling to invalid local minima. Next, all parameters were treated as fitting variables for the final fitting results.

During the fitting process for $V_g \neq 0$ V data, we adopted a constraint that the bulk (hole) carriers were insensitive to V_g because the electric field decays rapidly in bulk metallic crystals due to the carrier screening effect, unlike thin film semiconductor samples. Even if the V_g affects bulk carriers, the metallic samples have tremendous bulk carriers ($\sim 10^{20}$cm^{-3}), and thus the variation of carrier number induced by the gating is negligible (the tunable carrier density in the present method is typically less than $\sim 10^{14}$cm^{-2}/V [S3]). Therefore, the constraint of taking bulk hole carriers as constant during the gating is natural. Additionally, we confirmed that similar fitting results were obtained without this constraint, as shown in Fig. S2 (e,f). The sensitivity of electron carriers to the gating ensures that the electron carriers condense at the surface and the IL-gating tunes the surface carriers.

Supplementary section 3:Offset calibration of the resistivity in the gate-tuned transport measurements

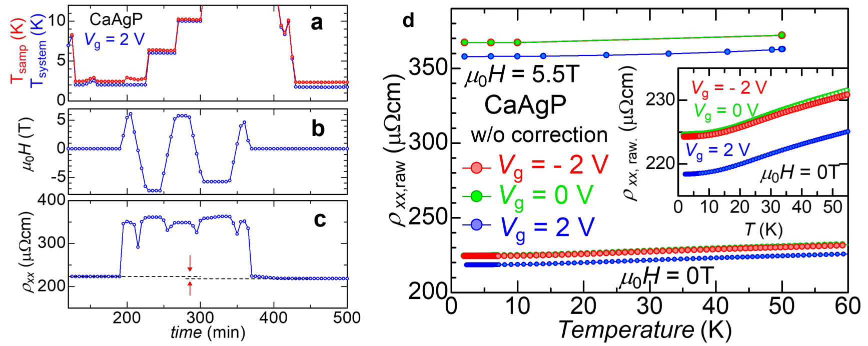

Fig. S3 Offset calibration of CaAgP resistivity. a–c, Time dependences of **a.** sample and system temperatures (T_{samp} and T_{system}), **b.** magnetic field ($\mu_0 H$), **c.** resistivity of CaAgP (ρ_{xx}) taken from measurement log data. The resistivity slightly changed while evaluating the magnetic field dependence of the resistivity. The red arrows indicate the resistivity change at 2 K compared with before/after the ρ–H measurements, probably due to some mechanical instability of the electrodes. **d,** Temperature dependence of raw resistivity of the gated CaAgP with/without the magnetic field. It shows the raw resistivity data of CaAgP without the resistivity correction for $V_g = 2$V.The inset highlighted the ρ–T at zero-field.

Figures S3 (a)–(c) show the logging data during the measurement. The ρ–T of CaAgP under $V_g = 2$ V shown in Fig. S3(c) exhibit a jump during transport measurement. The extrapolated curve of resistivity at 2K before and after ρ–T sweep and ρ–H measurement shows an apparent discrepancy as marked by the red arrow. Similar resistivity jumps were often observed in IL-gated transport measurements, and the resistivity tended to be returned to its original value after a certain duration. We consider that the jumps were induced mechanically due to the partial removal of IL from the sample surface by the thermal shrinking of the IL. Such the resistivity jump should be corrected properly because it is induced by an external origin other than electronic states. We corrected the resistivity by normalization (we multiplied the ratio of resistivity change for ρ–T data), as seen in Fig. S3(d). In warming the sample, we confirmed that the resistivity slightly increased in a stair-step-like around the nitrogen temperature and that the corrected resistivity at low temperatures follows higher-temperature ρ–T curves smoothly.

Note that the resistivity correction does not affect ρ–H and the fitting results in Fig. 1(e) and (f) because ρ–T measurement was the last measurement in the whole sequence, and the resistivity drop did not occur before ρ–H measurement at 2 K.

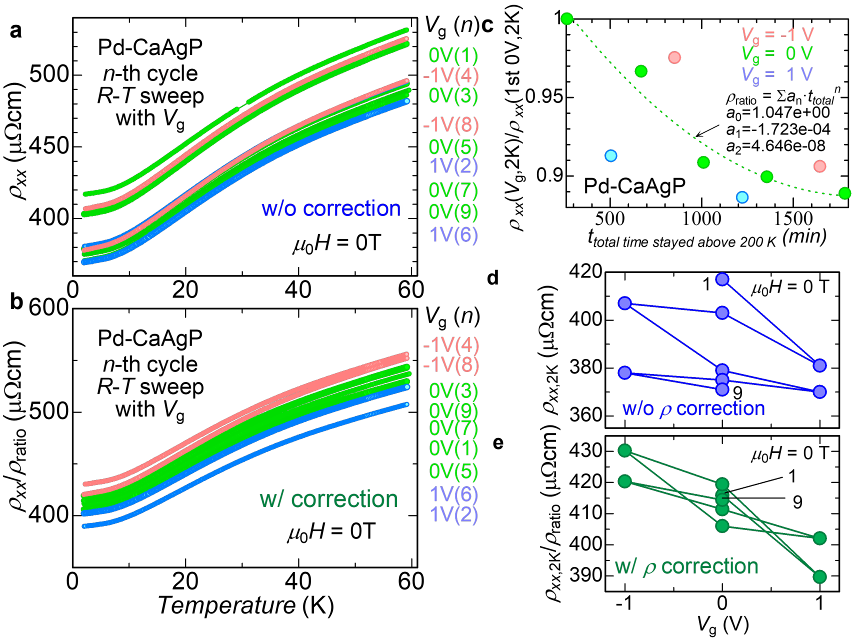

Fig. S4 Evaluation of the resistivity for Pd-CaAgP with the gate voltages and their cycle dependence. a, The temperature dependence of the resistivity at different gate voltages without a correction (a) and with a correction by normalization (b). **c,**, The total time (staying above 200 K) dependence of the resistivity at 2K. The dashed line represents a quadratic fitting curve based on $V_g = 0$ V data. **d, e**, Voltage dependence of the resistivity at 2 K without a correction (d) and with a correction (e). The numbers represent the cycle numbers.

In a preliminary measurement of gated transports, we observed that the resistivity changed during the temperature sweeping cycle at around nitrogen temperature and 200 K, which corresponds to the glass temperature of the IL. In addition, the sensing currents may induce electrochemical reactions in the liquid state of the IL. Thus, we performed all transport measurements of Pd-CaAgP below 60 K to avoid the abovementioned adverse effects. However, the resistivity slightly drifted possibly due to the instability of the electrodes during the cooling process, similar to undoped CaAgP.

We repeated the transport measurement for several sweep cycles to distinguish the gate dependence and cycle dependence. Figure S4(a) shows the data for all cycle measurements. The numbers in the bracket represent the cycle number. (the total cycles are 9, and we used the 6-9th cycle in the main text). We also replot the resistivity values at 2 K to find the general trend in Fig. S4(d). We found that the downward-sloping responses of the resistivity to the gate voltage were quite reproducible, as shown in Fig. S4(d). Additionally, we found resistivity drift components other than the gate voltage dependence. (the resistivity with the same gate voltage should have the same values in the ideal gate-tuned measurements.) The amplitude of the drift component

depended not on the total time or cycle numbers but on the total duration staying above 200 K (near the glass temperature of the ionic liquid (IL)). This fact implies that the IL gradually breaks the electrodes, and thus effective width of the electrodes may change. If the IL chemically reacted with the crystals, the resistivity should usually be enhanced due to oxidation on the metallic samples, and any reacted compounds should exist after the measurements. We did not find such reacted substances. Thus, we concluded that the instability of the electrodes cause the resistivity drift.

We tried to plot various plots to find best calibration method. As a result, we found the duration time spent above 200 K highly correlated with the resistivity value described above. To calibrate the drift components, we plot the normalized resistivity divided by the first cycle ($V_g = 0$V) resistivity at 2K as a function of the total duration staying above 200 K [Fig. S4(b)]. We obtained a quadratic fit curve for $V_g = 0$ V values. Based on this analysis, we corrected the resistivity shown in Fig. S4(c) and (e) and confirmed the clear gate dependence. To discuss the gate-dependence of the transports, we applied those corrected data in the main text (We used the 6-9th cycle in the main text).

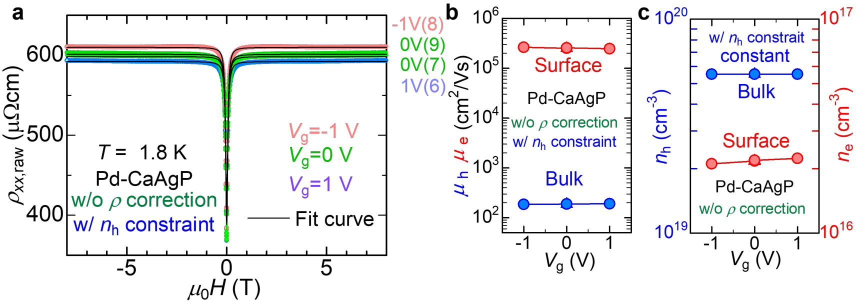

Fig. S5 The magnetic field dependence of the raw resistivity (without ρ correction) of Pd-CaAgP with 6-9 cycles. a The black lines represent the fitting results. **b,c,** The mobility (b) and carrier density at 2K determined by the fitting of the (a).

Using the data without the resistivity correction, we re-evaluated the gate dependence of the carrier density and mobility. Fig. S5(a) shows the field dependence of the resistivity without the correction for 6–9 sweep cycles. As a result of the two-band model fitting, we obtained the carrier density and mobility, considering the bulk carrier was unchanged [Fig. S5(b) and (c)]. These are almost the same as those obtained with ρ correction in Fig. 1(f).

We explicitly show all fitting results in Table. S1. The drift component influences the absolute value of the electron carrier density to be overestimated. However, the change in carrier density by the gate voltage does not change before and after the ρ correction. On the other hand, the constraint that nh is invariant also has almost no effect on the value of surface carrier density. Therefore, the conclusion that the surface electron carrier density varies with

Table S1 Summary of the obtained fitting results. Each table explicitly shows the fitting parameters of Fig. 1(f) [top panel], Fig. S2(f) [middle], and Fig. S5 (b,c) [bottom panel]. The two-band model fitting with/without a constrained condition (n_h regards as a constant) is performed for ρ–H curves with/without ρ correction (the normalization removes the drift components). The general trend is the same with or without ρ correction and with or without nh constraints.

**w/ ρ correction & w/ n_h constraint** [Fig.1(f)]				
Vg [n-th] (v)	n_h ($\times 10^{19}$ cm^{-3})	μ_h (cm^2/Vs)	n_e ($\times 10^{16}$ cm^{-3})	μ_e (cm^2/Vs)
-1 [8]	5.5580	165.62	1.8957	263426
0 [7]	5.5580	170.40	1.9816	251903
0 [9]	5.5580	167.98	1.9591	256804
1 [6]	5.5580	174.43	2.0566	246277

**w/ ρ correction & w/o n_h constraint** [Fig.S2(f)]				
Vg [n-th] (v)	n_h ($\times 10^{19}$ cm^{-3})	μ_h (cm^2/Vs)	n_e ($\times 10^{16}$ cm^{-3})	μ_e (cm^2/Vs)
-1 [8]	5.5621	165.50	1.8957	263427
0 [7]	5.5580	170.39	1.9816	251903
0 [9]	5.5573	167.98	1.9591	256805
1 [6]	5.5644	174.23	2.0566	246278

**w/o ρ correction & w/ n_h constraint** [Fig.S5(b,c)]				
Vg [n-th] (v)	n_h ($\times 10^{19}$ cm^{-3})	μ_h (cm^2/Vs)	n_e ($\times 10^{16}$ cm^{-3})	μ_e (cm^2/Vs)
-1 [8]	5.5280	185.28	2.1091	263341
0 [7]	5.5280	187.94	2.1734	251912
0 [9]	5.5280	188.83	2.1901	256807
1 [6]	5.5280	190.81	2.2364	246455

gate voltage is solid since the ρ correction and the constraint condition do not affect the general trend.

The obtained fitting parameters are consistent with previous data without IL [S2]. The change in carrier density by the gate controlling was about 8×10^{14}cm^{-3}. This value corresponds to 1×10^{13}cm^{-2} using the sample thickness, consistent with other gated systems on semiconductor materials (e.g., the induced carrier density of MoS_2 is $\sim 10^{14}$cm^2 [S3] ,$SrTiO_3$ $\sim 10^{13-14}$cm^{-2} [S3]. Those consistencies also ensure the validity of gated-transport experiments and data corrections.

Supplementary section 4:Temperature dependence of the magnetoresistance

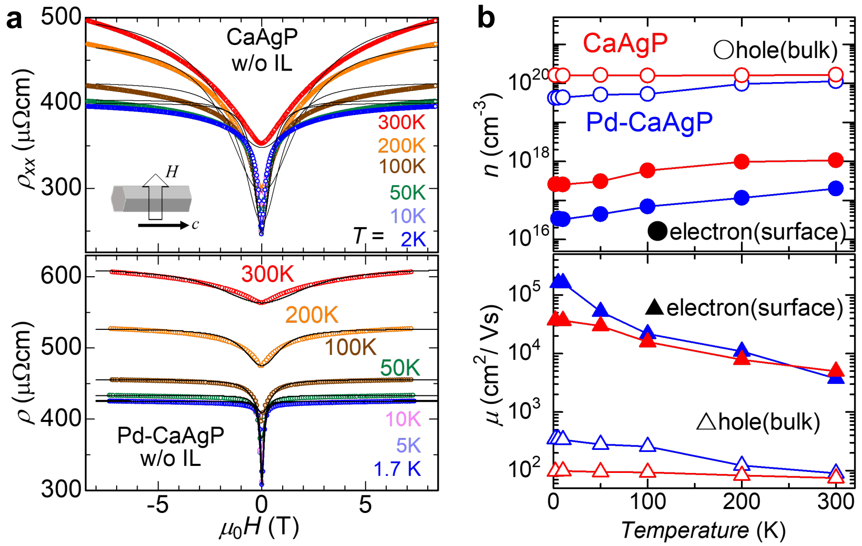

Fig. S6 Field-dependence of the resistivity in the perpendicular magnetic field at different temperatures for undoped CaAgP and Pd-CaAgP, and their analytical results by the two-band model. a, Resistivity change of $CaAg_{1-x}Pd_xP$ ($x =$ 0 for upper, and nominal 0.1 for bottom panels) with perpendicular magnetic field. Black lines are fitting results of the two-band model. **b,** The temperature dependence of the fitting results from the two-band model from (a). Filled and open symbols represent electrons and holes, respectively. The red and blue-colored symbols are results of CaAgP and Pd-CaAgP, respectively.

The temperature evolution of the resistivity was evaluated by the two-band model. The black lines in Fig. S6(a) represent the fitting result for each temperature. We obtained good fitting results for the whole temperature range. The obtained fitting parameters are summarized in Fig. S6(b). The temperature dependence of carrier density and mobility for the hole (bulk) in $CaAg_{1-x}Pd_xP$ shows typical metallic dependence (less affected by temperature), while those of electrons (surface) are semiconductive. This semiconductive dependence may reflect the Fermi energy beneath the flat surface band. In addition, this mobility temperature dependence seems to govern resistivity, significantly below 60 K.

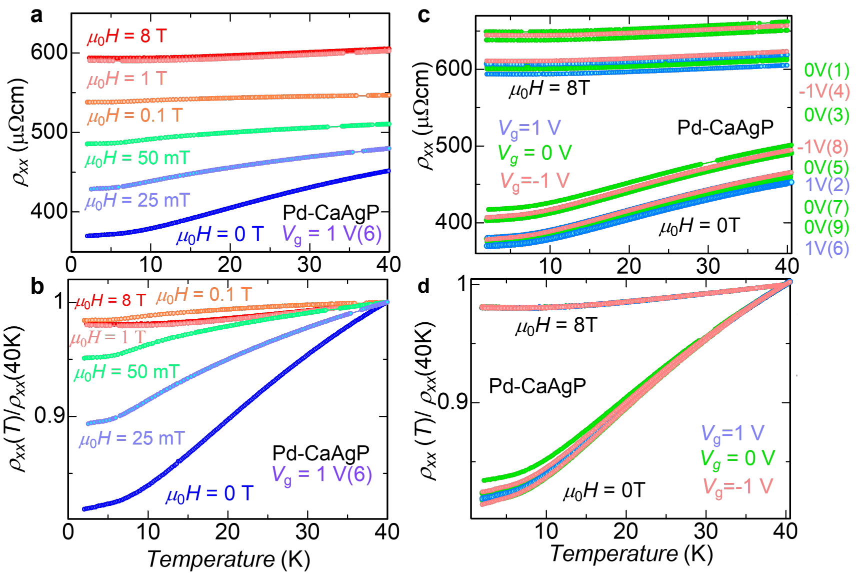

Fig. S7 The magnetic field effect for the gate-tuned ρ-T. a, b, Temerature dependence of resistivity under the different magnetic fields for Pd-CaAgP with the V_g=1 V (a) and their normalized resistivity by ρ_{xx}(40 K). **c, d,** Temerature dependence of resistivity at 0 and 8 T for Pd-CaAgP with different gate voltages (c) and their normalized resistivity by ρ_{xx}(40 K) (d).

We discuss the curious features of the magnetoresistance of Pd-CaAgP. Figure S7(a) shows typical examples of the temperature dependence of Pd-CaAgP with V_g = 1 V at different magnetic fields. Reflecting the magnetoresistivity, ρ-T changed by small magnetic fields and the curves beyond 1T show almost temperature independent below 15 K [Fig. S7(b)]. This general trend is valid for other gate voltages. While the absolute values of resistivity depend on the gate voltage [Fig. S7(c)], the normalized resistivity (divided by the value of 40 K) for the high magnetic field traces almost the same curve [Fig. S7(b, d)]. The magnetoresistance at high fields has the same temperature dependence for different gate voltages. This fact may be related to the origin of magnetoresistance and the band structures. Due to the extensive mobility of the electrons, the electrons should have a large cyclotron frequency ω and a large relaxation time of the carriers τ, and thus the high-field condition ($\omega\tau >> 1$, which is sufficient to form a complete cyclotron orbit, and may contribute to the saturation resistivity) is easily achieved by a comparably small field (around 1T). This explanation is consistent with the experimental observation of the quantum oscillations above 2 T. The temperature independence of the normalized resistivity up to 15 K indicates a large characteristic energy gap of $\hbar\omega$ larger than thermal energy $k_{\mathrm{B}}T$. This fact is also consistent with the emergence of quantum oscillations up to 20 K. Quantitative relaxation time can be discussed from the Dingle temperature obtained from the temperature dependence of the quantum oscillation analysis, which is planned as

future work. Furthermore, no gate dependence on the normalized resistivity also implies that there are no excess electronic bands around the Fermi energy, since such additional bands should contribute to the conductivity.

Supplementary section 5: FFT analysis of SdH oscillation

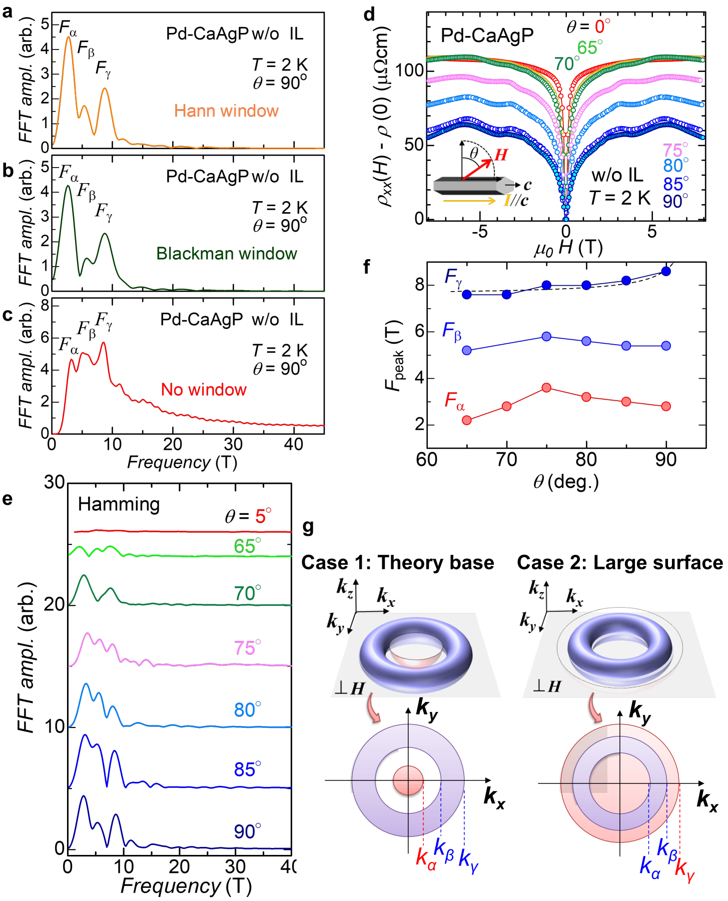

Fig. S8 FFT process dependence of SdH oscillations and their angular dependence for Pd-CaAgP. a–c, Window dependence of FFT amplitudes for SdH oscillations from Fig. 2(a) data. The windows used are (a) Hann, (b) Blackman windows, and (c) without windows. **d,** Angular dependence of magnetoresistance for $\theta = 0 - 90°$. The inset shows the measurement configuration. **e,** FFT analytical results with the Hamming window for SdH components of the data (d). **f,** Peak names are labeled from low frequency. The black dashed line represents $1/\cos\theta$ dependence. **g,** Possible two cases for Fermi surface states and their cross-section if three peaks correspond to the bulk and the surface. The $k_i (i = \alpha, \beta, \gamma)$ represents that calculated from correspondence peak frequency F_i. The Red colored circles and characters represent surface states.

Usually, FFT analysis is effective for data consisting of high-frequency components, while that for low frequency depends on analysis methods. First, we confirmed filtering window dependence, as shown in Fig. S8(a)–(c). All windows produce three peaks. At $\theta = 90°$, the values of $k_i (i = \alpha, \beta, \gamma)$ are 0.092, 0.012, and 0.016 Å^{-1}, respectively. Angular dependence of SdH (Fig. S8(d))

was evaluated using Hamming window, as shown in Fig. S8(e). The three peaks slightly show angular dependence within a limited small magnetic field and angle range. As a general trend, they slightly increase with increasing angle θ. One possible interpretation is that they behave as quasi-two-dimensional carriers, which follows $1/\cos\theta$. The black dashed line represents $1/\cos\theta$ dependence considering possible misalignment of several degrees. The main text discusses that the peaks may correspond to the surface states. However, if we consider two cases assuming a simplest torus Fermi surface with the surface states (Fig. S8(g)), one plausible interpretation is that the lowest peak corresponds to the surface states and the others form the torus, which produces the bulk carriers $n_{3\mathrm{D}} = 1.3 \times 10^{16}$ cm^{-3}. This value is several orders of magnitude smaller than n_{h} of the two-band fitting result. The other interpretation is that F_γ represents the surface state larger than the torus as a cross-section. Assuming this case 2, the carrier densities of the surface are $n_{2\mathrm{D}} = 2.1 \times 10^{11}$ cm^{-2}, comparable with the two-band model results with a surface thickness of several nanometers. The bulk carriers, however, become further small. In other words, those low-frequency peaks produce a sheet carrier density of 10^{10-12} cm^{-2}. This is the reason we concluded that all peaks represent surface states. Indeed, the hexagonal, needle-like crystals have three different crystal planes that potentially produce three peaks. Further experiments applying a large magnetic field and precise angular dependence are indispensable to determine the exact peak assignment.

Supplementary section 6: Low-temperature measurements for detail SC properties

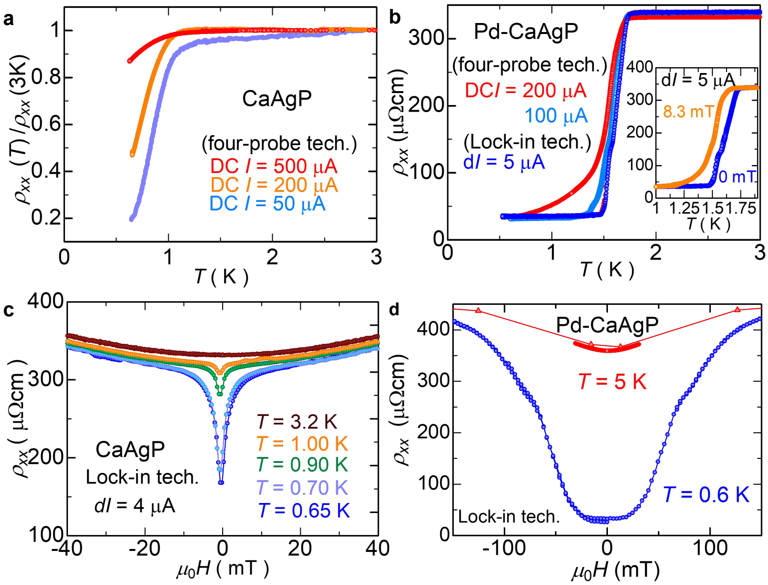

Fig. S9 Superconducting properties of undoped CaAgP and Pd-CaAgP a,b, Temperature-dependence of resistivity of $CaAg_{1-x}Pd_xP$ ($x = 0$ (a), and nominal 0.1 (b)) with different sensing currents. We applied the standard lock-in technique (applying small ac-current for measurements) to measure resistance with senseing current below 10 μA, while the standard dc four-probe measurements for 15–500 μA. The inset of (b) shows $\rho - T$ with a variation of the magnetic field. **c,d,** Field-dependence of resistivity of CaAgP (c) and Pd-CAgP (d) at different temperatures. Undoped CaAgP rapidly increases resistance and breaks superconductivity by a tiny magnetic field. SC for undoped CaAgP was destroyed by a small magnetic field like a remanent magnetic field $\sim$ several mT at 0.6 K.

Detail superconducting properties were evaluated. One of the unique features of SC in this system is the small critical current and magnetic fields. Figure S9 (a) and (b) show the temperature dependence of (normalized) resistivity with different measurement currents for undoped (a) and Pd-doped CaAgP (b). Due to the limitation of measurement equipment, we used two measurement techniques. We adopted the standard four-probe technique with large sensing currents, while we used the standard lock-in technique with small currents less than 10 μA, which adopts a small ac-current to acquire small signals. This technique often requires offset corrections due to the DC drift voltage derived from a preamplifier and thermal drift. Unfortunately, evaluating an exact offset value is difficult, and the voltage values are comparable to the offset voltage. Insufficient or over-calibration should affect the discussion of the 2D superconductivity. The R–T curve of Pd-CaAgP in Fig. 3 shows a slight temperature dependence even below T_c, while typical superconductors

show a flat temperature dependence due to zero resistivity. This finite non-zero resistivity should be related to the nature of 2D superconductivity at the surface. The temperature dependence is predicted to have a small exponential component just around T_{BKT}, as discussed in the ref. [S4]. Furthermore, as discussed later, $\mathrm{CaAg}_{1-x}\mathrm{Pd}_x\mathrm{P}$ has a high sensitivity to the sensing current. Therefore, the absolute value of the small resistivity is critical to discuss the nature of the 2D superconductivity. Here, we have refrained from calibrating the resistivity for low-temperature measurements below 3 K to avoid confusing discussion. We plot raw-resistivity data in Fig. 3 and Fig. S9. Given the normal resistivity values in Fig. S9(c) and (d), we can roughly estimate the resistivity shift due to the offset components by comparing the resistivity of the samples without IL (Fig. S6). The offset resistivity for CaAgP (Pd-CaAgP) is about 100 (40) $\mu\Omega$cm. We plan to measure such a small resistivity with sufficient confidence to discuss the nature of 2D superconductivity as a future work.

At the lowest measurement temperature of 0.6 K, the resistivity of CaAgP reached about 80% reduction from that of 3 K with small sensing currents. Although we did not observe a clear zero resistivity for undoped CaAgP, such a drastic resistivity change should be due to superconductivity, and other magnetic and current dependences of the resistivity are also consistent with the superconductivity.

Resistance measurements for metallic samples usually adopt a large measurement current (typically I >1 mA). On the other hand, this material has a small critical current $I_{\mathrm{c1}} \sim 5$ μA, as shown in Fig. S9(a) and (b), which implies small critical magnetic fields. Figure S9(c) and (d) show resistivity versus magnetic fields at 0.6 K and normal state temperatures. For the measurement of CaAgP, a small measurement current was used for a magnetic field range of ± 20 mT. CaAgP (Pd-CaAgP) has second critical field of $H_{\mathrm{c2}} \sim 7$ mT (120 mT). Those critical fields are comparable with that of other low-carrier superconductors having similar T_{c}; for instance, Tl-doped PbTe with $T_{\mathrm{c}} = 1.4$ K has $H_{\mathrm{c2}} = 500$ mT at 0.6 K [S5].

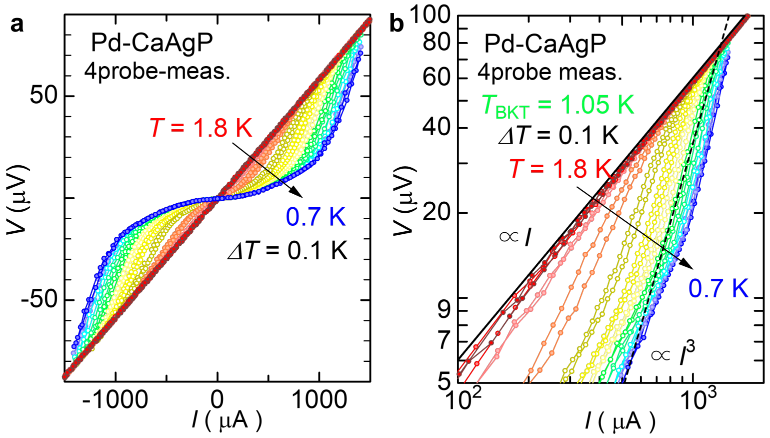

Fig. S10 Quasi-two-dimensional nature of superconductivity in Pd-CaAgP. a,b, $V-I$ curves at various temperatures from 1.8 K to 0.7 K with (a) standard scale and (b) log-log scale. The solid black line and the dashed black line represent the power law of $V \propto I$ and $V \propto I^3$, respectively. T_{BKT} is determined to be 1.05 K.

The voltage-current (V-I) curves of Pd-CaAgP showed a crossover behavior due to the Berezinskii-Kosterlitz-Thouless (BKT) transition for the two-dimensional superconductivity. Figure S10 (a) shows the V-I curves of the Pd-CaAgP crystal at various temperatures. The V-I curves change from linear into non-linear dependence at $T_{\mathrm{c}} = 1.7$ K. Due to low critical currents, the sample shows zero resistance with a narrow range of $\sim$100 μA. We plot the same data on the log-log scale in Fig. S6 (b). While V-I curves follow a typical Ohmic dependence ($V \propto I$) at high temperature, they show a smooth transition [$V \propto I^{\alpha}$ with the slope α exceeding $\alpha = 3$ (the dashed black line) from $\alpha = 1$ (the dashed line)]. This type of crossover is called the BKT transition. Similar smooth transitions were widely observed in other two-dimensional (2D) SC (magic angle bilayer [S6] and MoS_2 [S7]), while a theory predicts jump discontinuity for the infinite system. Other groups pointed out that this discrepancy may come from a finite-size effect [S8]. Thus, observing the BKT transition in Pd-CaAgP indicates the emergence of the 2D SC, which supports the surface SC.

Supplementary section 7: BTK model for differential conductance of the conventional SC and *p*-wave SC

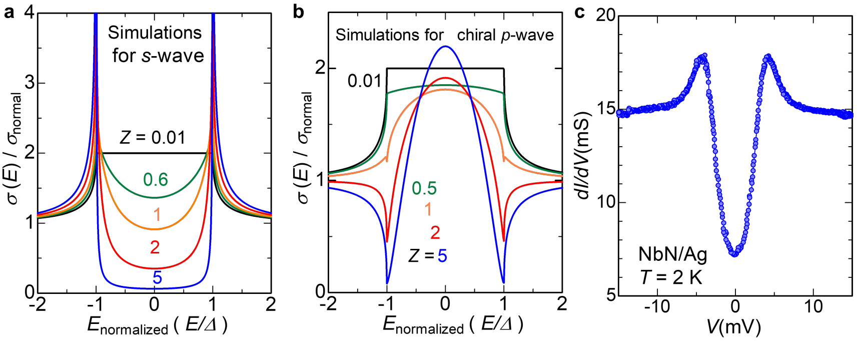

Fig. S11 Simulated spectra of typical *s*-wave and chiral *p*-wave superconductivity. a,b, Barrier potential-dependence of differential conductance spectra for *s*-wave (a), and chiral *p*-wave superconductivity (b) simulated by the typical BTK model. The parameter Z represents dimensionless barrier height as $Z = \frac{k_F H}{2E_F} = \frac{H}{\hbar \mu_F}$, where H is a repulsive potential. **c,** Experimental example for soft point contact spectrum of NbN as a typical *s*-wave superconductor at 2 K.

The soft point contact technique can probe the DOS of quasiparticles via quasiparticle tunneling by measuring differential conductance spectra [S9]. These spectra also depend on barrier potential at the interface between the superconductor-normal metal. Figure S11(a) and (b) show the barrier potential-dependence of normalized conductance for *s*-wave and *p*-wave superconductors, simulated within an extended BTK formula [S10, S11, S12]. For low barrier cases, normalized conductance shows almost doubled values due to excess current derived from Andreev reflection. The conductance reflects the DOS of quasiparticles when the barrier is high e.g., $Z > 1$. A typical *s*-wave superconductor shows a superconducting gap within $-\Delta < E < \Delta$ and sharp peaks at $E = \Delta$. On the other hand, a chiral *p*-wave superconductor shows a broad peak within the $-\Delta < E < \Delta$ and unique dips at $E = \Delta$. These dips are the hallmark of an unconventional superconductor because the *s*-wave superconductor does not show dips at $E = \pm\Delta$ even for low barrier cases [S9]. Figure S11(c) shows an experimental example for soft point contact spectroscopy to probe NbN/Ag junctions on NbN thin film 2 K. NbN is a typical *s*-wave superconductor, and thus, the spectrum shows a clear gap structure with superconducting gap $\Delta = 4$ meV. In addition to the above spectra, Majorana bound states (zero energy Andreev bound state) forms a sharp zero-bias conductance peak due to particle-antiparticle symmetry. Several unconventional SC, such as chiral *p*-wave SC, can host Majora Fermions (details can be found in ref in [S12, S13]. They are expected to emerge at the edge states and center of a vortex. The contacts of the present soft point contact measurements contain the edge of the crystals, and thereby it can potentially detect

the Majorana Fermions if they emerge. The sharp zero-bias peak also emerges at T_c from the temperature dependence of the conductance spectra [Fig. 4(d)]. Those behaviors can be understood as the emergence of the Majorana particles at the crystal edge or trapped vortex. On the other hand, several other mechanisms, such as disorders and weak antilocalization, also produce a zero-bias peak in conductance spectra [S14]. Detailed experiments to identify its origin should be performed in the future.

A spurious zero-bias conductance peaks (ZBCP) can emerge when the point-contact junction is in the thermal regime (the cases of highly transparent junctions) described by Sheet *et al.* [S15]. The local tunneling current exceeding J_{c1} of the local area induces the transition to a finite resistance state, which produces the heating effect. As a result, the conductance reduction yields a spurious ZBCP in $\mathrm{d}I/\mathrm{d}V$ as a function of the bias current.

We rejected the influence of the heating effect based on the reasons listed below. First, we estimated the J_{c1} of the surface SC and compared the bias current density. From the V-I curves (Fig. S10), we evaluated the J_{c1}, that the resistance deviates from zero, and J_{c2}, that the resistance reaches the normal state value. The estimated values of J_{c1} and J_{c2} were $1.7 \times 10^3 \mathrm{A/cm}^2$ and $7.4 \times 10^4 \mathrm{A/cm}^2$, respectively, assuming the thickness of the surface SC is 5 nm (because the thickness is assumed as several nanometers in the manuscript, which producing 2D carrier density of $\sim 10^{11} \mathrm{cm}^{-2}$). On the other hand, the bias current density at the point contact was up to $\sim 5 \mathrm{A/cm}^2$, which is several orders smaller than the J_{c1} and J_{c2}. This fact indicates that the injection current has negligible influence on the superconductivity.

Second, if the conductance spectrum features are due to the heating effect, the gap amplitude does *not* reflect the correct Δ_0. On the other hand, the detected value of the $2\Delta_0/k_{\mathrm{B}}T_{\mathrm{c}} = 5.97$ [the Δ_0 determined by the location of the dip in the $\mathrm{d}I/\mathrm{d}V$ spectra in Fig. 4(c)] is a reasonable value for unconventional superconductivity suggested in the Uemura plots.

Third, we checked the conductance spectra of the point contact junctions under small magnetic fields. If the heating effect mainly contributes to the ZBCP, the zero-bias conductance ($\mathrm{d}I/\mathrm{d}V$ at $V = 0$) should remain *unchanged* in the magnetic field below H_{c1}, because the junction resistance at the zero-bias is determined by the resistance of the superconductor and the normal metal in the thermal regime [S15]). The conductance is expected to show discontinuity when the applied field exceeds H_{c1}. On the other hand, when the conductance spectra reflect the density of states, the height of the ZBCP is determined by the thermal smearing effect of the Fermi distribution function. In such cases, the magnetic field response is continuous because the peak height is dominated by the ratio of temperature to the gap amplitude. To check this feature, we fabricated two junctions on the top/ side of the Pd-CaAgP crystal (the top plane is the (0 0 1) plane, and the side is the (1 0 0) plane). The magnetic field was applied along the c-axis (thereby, the top junction should rapidly degrade by the magnetic field reflecting the two-dimensional superconductivity). The evaluated H_{c1} of Pd-CaAgP is approximately 20 mT, as shown in Fig. S9(d)

We observed broad ZBCPs and dips for both side and top junctions similar to Fig. 4(d) in the manuscript. To see a small magnetic field effect below H_{c1}, we plotted magnetic field dependence of the conductance value at zero-bias (not shown here). It is clear that the zero-bias conductance for both junctions continuously responded from the zero magnetic fields, which indicates that the heating effect does not dominate the ZBCP.

Based on all considerations, we concluded that the conductance spectra reflect the density of states of the quasiparticles of unconventional SC, such as the chiral p-wave SC, not come from the heating effect.

References

[S1] Xu, N. *et al.* Trivial topological phase of CaAgP and the topological nodal-line transition in CaAg($P_{1-x}As_x$). *Phys. Rev. B* **97**, 161111(R) (2018).

[S2] Okamoto, Y. *et al.* High-mobility carriers induced by chemical doping in the candidate nodal-line semimetal CaAgP. *Phys. Rev. B* **102**, 115101 (2020).

[S3] Ueno K. *et al.*, Field-Induced Superconductivity in Electric Double Layer Transistors. *J. Phys. Soc. Jpn.* **83**, 032001 (2014).

[S4] Halperin, B. I. & Nelson, D. R., Resistive transition in superconducting films. *J Low Temp Phys* **36**, 599616 (1979).

[S5] Matsushita, Y., Wianecki, P. A., Sommer, A. T., Geballe, T. H. & Fisher, I. R. Type II superconducting parameters of Tl-doped PbTe determined from heat capacity and electronic transport measurements. *Phys. Rev. B* **74**, 134512 (2006).

[S6] Cao, Yuan, Fatemi, Valla, Fang, Shiang, Watanabe, Kenji, Taniguchi, Takashi, Kaxiras, Efthimios, and Jarillo-Herrero, Pablo Unconventional superconductivity in magic-angle graphene superlattices. *Nature* **556**, 4350 (2018). https://doi.org/10.1038/nature26160 .

[S7] J. M. Lu and O. Zheliuk, I. Leermakers, N. F. Q. Yuan, U. Zeitler, K. T. Law, and J. T. Ye, Evidence for two-dimensional Ising superconductivity in gated MoS_2, *Science* **350**, 1353-1357 (2015). https://www.science.org/doi/abs/10.1126/science.aab2277

[S8] Medvedyeva, Kateryna, Kim, Beom Jun and Minnhagen, Petter, Analysis of current-voltage characteristics of two-dimensional superconductors: Finite-size scaling behavior in the vicinity of the Kosterlitz-Thouless transition, *Phys. Rev. B* **62**, 14531–14540 (2000). https://link.aps.org/doi/10.1103/PhysRevB.62.14531

[S9] Daghero, D. & Gonnelli, R. S. Probing multiband superconductivity by point-contact spectroscopy. *Supercond. Sci. Technol.* **23**, 043001 (2010).

[S10] Yamashiro, M., Tanaka, Y. & Kashiwaya, S. Theory of tunneling spectroscopy in superconducting Sr_2RuO_4. *Phys. Rev. B* **56**, 7847–7850 (1997).

[S11] Kashiwaya, S. & Tanaka, Y. Tunnelling effects on surface bound states in unconventional superconductors. *Reports Prog. Phys.* **63**, 1641–1724 (2000).

[S12] Blonder, G. E., Tinkham, M. & Klapwijk, T. M. Transition from metallic to tunneling regimes in superconducting microconstrictions: Excess current, charge imbalance, and supercurrent conversion. *Phys. Rev. B* **25**, 4515–532 (1982).

[S13] Tanaka, Y. & Tamura, S. Theory of Surface Andreev Bound States and Odd-Frequency Pairing in Superconductor Junctions. *J. Supercond. Nov. Magn.* **34**, 1677–1694 (2021).

[S14] Devillard, P., Chevallier, D. & Albert, M. Fingerprints of Majorana fermions in current-correlation measurements from a superconducting tunnel microscope. *Phys. Rev. B* **96**, 115413 (2017).

[S15] Sheet, Goutam, Mukhopadhyay, S., and Raychaudhuri, P., Role of critical current on the point-contact Andreev reflection spectra between a normal metal and a superconductor, *Phys. Rev. B* **69**, 134507 (2004). https://link.aps.org/doi/10.1103/PhysRevB.69.134507
